# Restricted feeding modulates peripheral clocks and nutrient sensing pathways in rats

**DOI:** 10.20945/2359-3997000000407

**Published:** 2021-09-29

**Authors:** Luis Guilherme F. Rodrigues, Leonardo D. de Araujo, Silvia L. R. Roa, Ana C. Bueno, Ernane T. Uchoa, José Antunes-Rodrigues, Ayrton C. Moreira, Lucila L. K. Elias, Margaret de Castro, Clarissa S. Martins

**Affiliations:** 1 Universidade de São Paulo Faculdade de Medicina de Ribeirão Preto Departamento de Medicina Interna Ribeirão Preto SP Brasil Departamento de Medicina Interna, Faculdade de Medicina de Ribeirão Preto, Universidade de São Paulo, Ribeirão Preto, SP, Brasil; 2 Universidade de São Paulo Faculdade de Medicina de Ribeirão Preto Departamento de Fisiologia Ribeirão Preto SP Brasil Departamento de Fisiologia, Faculdade de Medicina de Ribeirão Preto, Universidade de São Paulo, Ribeirão Preto, SP, Brasil; 3 Universidade de São Paulo Faculdade de Medicina de Ribeirão Preto Departamento de Pediatria Ribeirão Preto SP Brasil Departamento de Pediatria, Faculdade de Medicina de Ribeirão Preto, Universidade de São Paulo, Ribeirão Preto, SP, Brasil

**Keywords:** Caloric restriction, feeding behavior, biological clocks, circadian rhythm, sirtuin 1

## Abstract

**Objective:**

Feeding restriction in rats alters the oscillators in suprachiasmatic, paraventricular, and arcuate nuclei, hypothalamic areas involved in food intake. In the present study, using the same animals and experimental protocol, we aimed to analyze if food restriction could reset clock genes ( *Clock, Bmal1* ) and genes involved in lipid metabolism ( *Pgc1a, Pparg, Ucp2* ) through nutrient-sensing pathways ( *Sirt1, Ampk, Nampt* ) in peripheral tissues.

**Materials and methods:**

Rats were grouped according to food access: Control group (CG, food *ad libitum* ), Restricted night-fed (RF-n, food access during 2 h at night), Restricted day-fed (RF-d, food access during 2 h in the daytime), and Day-fed (DF, food access during 12 h in the daytime). After 21 days, rats were decapitated at ZT3 (0900-1000 h), ZT11 (1700-1800 h), or ZT17 (2300-2400 h). Blood, liver, brown (BAT) and peri-epididymal (PAT) adipose tissues were collected. Plasma corticosterone and gene expression were evaluated by radioimmunoassay and qPCR, respectively.

**Results:**

In the liver, the expression pattern of *Clock* and *Bmal1* shifted when food access was dissociated from rat nocturnal activity; this phenomenon was attenuated in adipose tissues. Daytime feeding also inverted the profile of energy-sensing and lipid metabolism-related genes in the liver, whereas calorie restriction induced a pre-feeding increased expression of these genes. In adipose tissues, *Sirt1* expression was modified by daytime feeding and calorie restriction, with concomitant expression of *Pgc1a* , *Pparg* , and *Ucp2* but not *Ampk* and *Nampt* .

**Conclusion:**

Feeding restriction reset clock genes and genes involved in lipid metabolism through nutrient-sensing-related genes in rat liver, brown, and peri-epididymal adipose tissues.

## INTRODUCTION

Biological circadian rhythms allow organisms to prepare for recurrent daily changes in the light-dark cycle and food availability. In mammals, the circadian rhythms are controlled by the circadian clock system, comprising a central pacemaker localized in the suprachiasmatic nucleus (SCN) that synchronizes peripheral pacemakers in different tissues. The circadian clock system consists of a complex of transcriptional-translational feedback loops regulated by the “clock genes.” In the main loop, the heterodimeric transcription factors CLOCK and BMAL1 promote the transcription of their inhibitors, the *Cryptochromes* ( *Cry1* , *Cry2* ) and *Periods* ( *Per1* , *Per2* and *Per3)* genes. *Bmal1* and *Clock* genes also increase the mRNA levels of *Rev-erba* and *Rora* , which compete for binding to the retinoic acid-related orphan receptor response elements (ROREs) repressing or activating the expression of *Bmal1* ( 1 ). The circadian clocks are self-sustained, but they are synchronized by external zeitgebers. Whereas the SCN is synchronized mainly by photic stimuli, the peripheral oscillators also respond to other cues, such as feeding pattern and temperature ( 1 , 2 ).

The molecular circadian clock is an important regulator of metabolism and feeding behavior. Moreover, feeding time affects the circadian clock. *Clock* and *Rev-erba* mutant mice develop obesity ( 3 , 4 ). However, obesity is avoided in *Clock* mutant mice submitted to time-restricted feeding, even without altering levels of food consumption or activity ( 5 ).

In mice, daytime feeding is capable of uncoupling the peripheral oscillators from the SCN ( 6 ). In humans, night shift workers present increased risk of type 2 diabetes mellitus (T2DM) and metabolic syndrome (MetS) compared with day shift workers ( 7 ). Currently, animal models have been used to mimic shiftwork conditions. Studies using these animals have enabled researchers to evaluate the consequences of circadian disruption ( 8 ) and possible mechanisms responsible for metabolic disease development. Moreover, they allow researchers to evaluate the arising potential therapies for MetS and T2DM targeting the circadian molecular clock, such as REV-ERB agonists, ROR agonists, and CRY stabilizers ( 7 ).

To date, the mechanisms responsible for the resetting of peripheral clocks in response to changes in feeding time are not completely understood. Glucocorticoids are plausible candidates because they are secreted in daily cycles and are able to modulate the expression of clock genes causing phase shifts exclusively in peripheral cells ( 9 ). The importance of the glucocorticoid receptor for the regulation of clock genes by feeding entrainment was recently demonstrated in zebrafish, a non-mammalian species ( 10 ). In adrenalectomized mice submitted to daytime feeding, larger phase shifts were observed compared with controls, suggesting that glucocorticoids in fact counteract the re-phasing of peripheral clocks ( 11 ).

Nutrient-sensing molecules, such as SIRT1 (sirtuin-1) and AMPK (AMP-activated protein kinase), act as clock resetting signals ( 12 ). SIRT1 is a deacetylase, which requires the coenzyme NAD^+^. The NAD^+^/NADH status serves as a surrogate marker for the cellular redox state. According to the redox status, SIRT1 targets key metabolic proteins and can deacetylase histones, possibly counteracting the histone acetyltransferase activity of CLOCK ( 13 ). Furthermore, SIRT1 promotes deacetylation of important clock machinery proteins: the circadian repressor PER2, leading to its degradation, and BMAL1, which is recruited by the CLOCK/BMAL1 complex, consequently regulating gene expression ( 12 ).

AMPK is a heterotrimeric enzyme with a glycogen-binding domain and sites for the binding of AMP, ADP, and ATP. In fact, its activity is increased by ATP depletion, and some of the clock system components contain potential AMPK target phosphorylation sites ( 14 ). SIRT1 and AMPK interact with each other and might also regulate different metabolic pathways, including lipid storage, synthesis, and usage. AMPK also influences SIRT1 by increasing cellular nicotinamide phosphoribosyl-transferase (NAMPT) expression, thereby increasing NAD^+^ levels and consequently SIRT1 activity ( 15 ). Therefore, SIRT1 and AMPK may be capable of transducing signals related to feeding inputs to the clock machinery.

In a previous study from our lab, we demonstrated that the master oscillator in the SCN – as well as the oscillator in paraventricular and arcuate hypothalamic nuclei, brain areas involved in food intake – responds in a tissue-specific manner to feeding restriction in rats ( 16 ). In the present study, we took advantage of the same animals submitted to that experimental protocol and hypothesized that different feeding restriction patterns could reset clock genes and genes involved in lipid metabolism ( *Pgc1a* , *Pparg* , *Ucp2* ) through nutrient-sensing-related genes ( *Sirt1* , *Ampk* , *Nampt* ) in rat peripheral tissues, such as liver, brown, and peri-epididymal adipose tissues.

## MATERIALS AND METHODS

### Animals and housing

**Figure 1:**
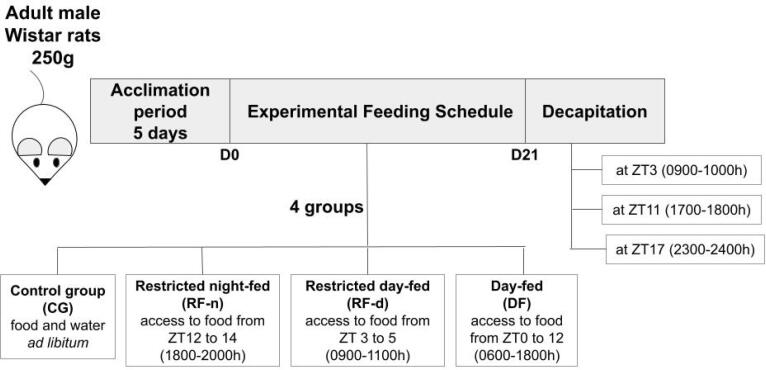
Schematic design of the experiment.

This study was approved by the Animal Ethics Committee of the Ribeirao Preto Medical School of the University of Sao Paulo, Brazil (Protocol n° 077/2011). The animals used in the present study were submitted to the experimental procedure performed in our lab and previously published ( 16 ). Briefly, young adult male Wistar rats aged approximately 8 weeks, weighing between 200 g and 300 g (average weight = 250 g), obtained from the Animal Facility of the Ribeirao Preto Medical School, were housed for 5 days in individual hanging wire cages with food and water *ad libitum* , under controlled conditions of temperature (23 °C with a maximum variability range of 4 °C) and humidity (range between 40 and 60%). The light was regulated on a 12/12 h light/dark cycle, with lights on at 6:00 A.M. (zeitgeber time [ZT] 0). All animals were fed with standard chow. As shown in Figure 1 , after five days of acclimation, the rats were divided into four groups with different dietary patterns for 20 days – Control group (CG, total of 16 rats): food and water *ad libitum* ; Restricted night-fed (RF-n, total of 19 rats): access to food from ZT12 to 14 (1800-2000 h); Restricted day-fed (RF-d, total of 22 rats): access to food from ZT3 to 5 (0900-1100 h); and Day-fed (DF, total number of 21 rats): access to food from ZT0 to ZT12 (0600-1800 h) – as previously published by our group ( 16 ). To avoid unspecific or stress-related elevations of corticosterone secretion, animals were handled by the same investigator during the experiment, and, on the last day, animals were decapitated within 60 seconds. Animals of the control group decapitated at ZT3, which showed plasma concentrations of corticosterone above 3 ug/dL, were excluded because of undesirable stress conditions, as previously published by our laboratory ( 17 ).

D: Day of experiment; ZT: zeitgeber time.

### Experimental design

Body weight and food intake were measured daily throughout the experiment. On the 21st day, rats were decapitated per time point at ZT3 (0900-1000 h), ZT11 (1700-1800 h), or ZT17 (2300-2400 h).

Trunk blood was collected for plasma corticosterone measurement. Tissues (liver, brown adipose tissue [BAT], and peri-epididymal adipose tissue [PAT]) were collected, flash-frozen in dry ice, and stored at -80 °C until RNA isolation.

### Corticosterone assay

Plasma corticosterone (B) was measured by radioimmunoassay as previously described ( 16 ). The assay sensitivity was 0.4 μg/dL, and the inter- and intra-assay variations were 4.8% and 6.7%, respectively.

### RNA isolation, cDNA synthesis and amplification, and real-time PCR

RNA from the liver, BAT, and PAT obtained from animals submitted to the previous experimental protocol ( 16 ) was isolated using TRIzol^®^ reagent (Invitrogen, Life Technologies, Carlsbad, CA, USA). Sample integrity was evaluated by spectrophotometry at an absorbance of 260/280 nm using NanoDrop^TM^ 2000/2000c (Thermo Fisher Scientific, Wilmington, Delaware, USA) and by agarose gel electrophoresis.

cDNA was obtained using the High-Capacity cDNA Reverse Transcription Kit (Applied Biosystems, Life Technologies, Foster City, CA, USA). qPCR was performed by the 7500 RT-PCR System (Applied Biosystems, Life Technologies, Foster City, CA, USA) using TaqMan^®^ Gene Expression Assays: *Clock* ( *Rn00573120_m1* ), *Bmal1* ( *Rn00577590_m1* ), *Sirt1* ( *Rn01428096_m1* ), *Ampk* ( *Rn00576935_m1* ), *Nampt* ( *Rn00822046_m1* ), *Pparg* ( *Rn00440945_m1* ), *Pgc1a* ( *Rn00580241_m1* ), *Ucp2* ( *Rn01754856_m1* ), and reference genes *Gapdh* ( *4352338E* ) and *Actb* ( *4352340E* ). Reactions were incubated in a 96-well optical plate at 95 °C for 10 min, followed by 40 cycles of 95°C for 15 sec and 60 °C for 1 min. The cycle threshold (Ct) was defined as the fractional cycle number at which the fluorescence surpasses the fixed threshold. Data were presented as the Ct-mean of each sample of each target gene normalized by the reference genes Ct-mean expression and calibrated by the ΔCt-median value obtained from animals decapitated at ZT3 (0900 h) of the control group. Relative expression was calculated using the 2^−ΔΔCt^ method, as previously detailed ( 16 ).

### Statistical analysis

Continuous variables were expressed as mean and standard deviation. Kruskal-Wallis with Dunn’s post-test was used for continuous variables. Data were analyzed by GraphPadPrism *5* software (GraphPad, San Diego, CA), and differences were considered significant at p < 0.05.

## RESULTS

In a previous study from our lab, rats were submitted to different restricted feeding schedules during light or dark phases to address clock gene expression in the SCN and other hypothalamic nuclei ( 16 ). Material collected from rats of that protocol were used for a gene expression study in the liver, BAT, and PAT.

### Body weight, food intake, and corticosterone measurement

Data on body weight, food intake, and corticosterone levels were published in a previous work from our lab ( 16 ). Briefly, all groups presented similar body weight at the beginning of the experiment. After 21 days, we observed a decreased in body weight (g) in the RF-n (228.1 ± 29.24) and RF-d (223.9 ± 44.43) groups compared with the CG (370.4 ± 47.95) and DF (369.6 ± 36.55) groups (p < 0.0001). Similarly, lower daily food intake (g) was observed in the RF-n (13.01 ± 1.14) and RF-d (13.52 ± 1.75) groups compared with the CG (31.05 ± 3.12) and DF (24.72 ± 2.82) groups (p < 0.0001). Consequently, lower caloric intake (kcal) was observed in the RF-n (51.74 ± 4.54) and RF-d (53.74 ± 6.96) compared with the CG (123.4 ± 12.42) and DF (98.28 ± 11.23) groups (p < 0.0001). In addition, the CG and RF-n groups presented higher corticosterone levels at ZT11 and ZT17 compared with ZT3, with no difference between ZT3 and ZT17. On the other hand, the RF-d group showed an inverted daily pattern of corticosterone secretion compared with the CG and RF-n groups, with higher levels at ZT3, whereas the DF group showed higher corticosterone levels at ZT11.

### Gene expression

Data on the gene expression of *Clock* , *Bmal1* , *Sirt1* , *Ampk* , *Nampt* , *Pgc1a* , *Pparg* , and *Ucp2* obtained from the liver, BAT, and PAT are presented as mean ± SD, median, and interquartile interval in Supplementary Tables 1 , 2 , and 3 , respectively.

**Figure 2:**
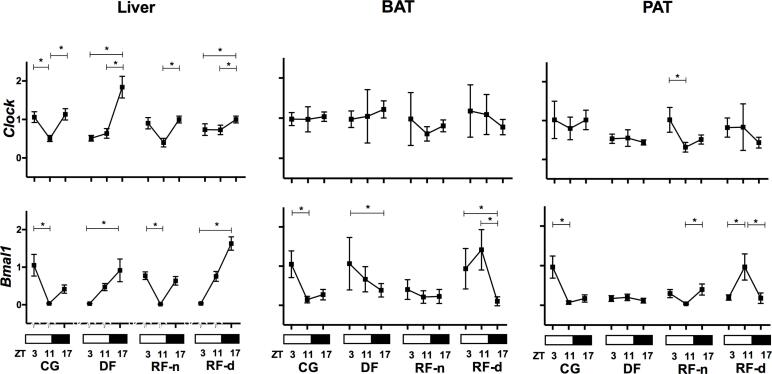
*Clock* and *Bmal1* gene expression (2^-ΔΔCt^) in the liver, BAT (brown adipose tissue), and PAT (peri-epididymal adipose tissue) of Control group (CG), Day-fed (DF), Restricted night-fed (RF-n), and Restricted day-fed (RF-d) rats at ZT3 (0900 h), ZT11 (1700 h), and ZT17 (2300 h).

Figure 2 shows the expression of *Clock* and *Bmal1* in the different tissues of the studied groups. In the CG, lower expressions of *Clock* (p = 0.0003) and *Bmal1* (p < 0.0001) were observed in the afternoon (ZT11) in the liver. This pattern was also seen for *Bmal1* in the PAT (p < 0.0001) and BAT (p < 0.0001), whereas *Clock* presented no difference among morning, afternoon, and night in BAT and PAT. In the RF-n group, lower expressions of *Clock* (p = 0.0006 and p < 0.0001) and *Bmal1* (p < 0.0001 and p = 0.0003) were observed in the afternoon (ZT11) in the liver and PAT, but not in the BAT. In the liver, the RF-d group showed an altered profile compared with the CG, with a lower expression of *Clock* (p = 0.002) and *Bmal1* (p < 0.0001) in the morning (ZT3) and a higher expression at night (ZT17), whereas higher expression of *Bmal1* was observed in the afternoon (ZT11) in the PAT and BAT (p = 0.0003). In contrast, in the PAT and BAT, no difference among morning, afternoon, and night was observed for *Clock* expression. Similar to the RF-d, the DF group also presented a lower expression of *Clock* (p = 0.0003) and *Bmal1* (p < 0.0001) in the liver in the morning (ZT3) and a higher expression at night (ZT17). In the BAT, this profile was inverted, with lower expression of *Bmal1* (p = 0.03) at night, whereas *Clock* presented no differential expression among ZT3, ZT11, and ZT17 in the PAT and BAT. Therefore, in the liver, food restriction during the night (RF-n) preserved the pattern of *Clock* and *Bmal1* expression observed in controls (CG), but when the food access occurred in dissociation with the rat’s nocturnal activity (RF-d and DF), we observed a shift of this profile. In the adipose tissues, we observed the same phenomenon at a lower magnitude. In rats submitted to daytime feeding (RF-d in BAT, RF-d and DF in PAT), the *Bmal1* expression pattern observed in the controls was lost or inverted. In addition, calorie restriction *per se* (RF-n) modified *Bmal1* expression in the BAT, but not in the PAT.

* p < 0.05

**Figure 3:**
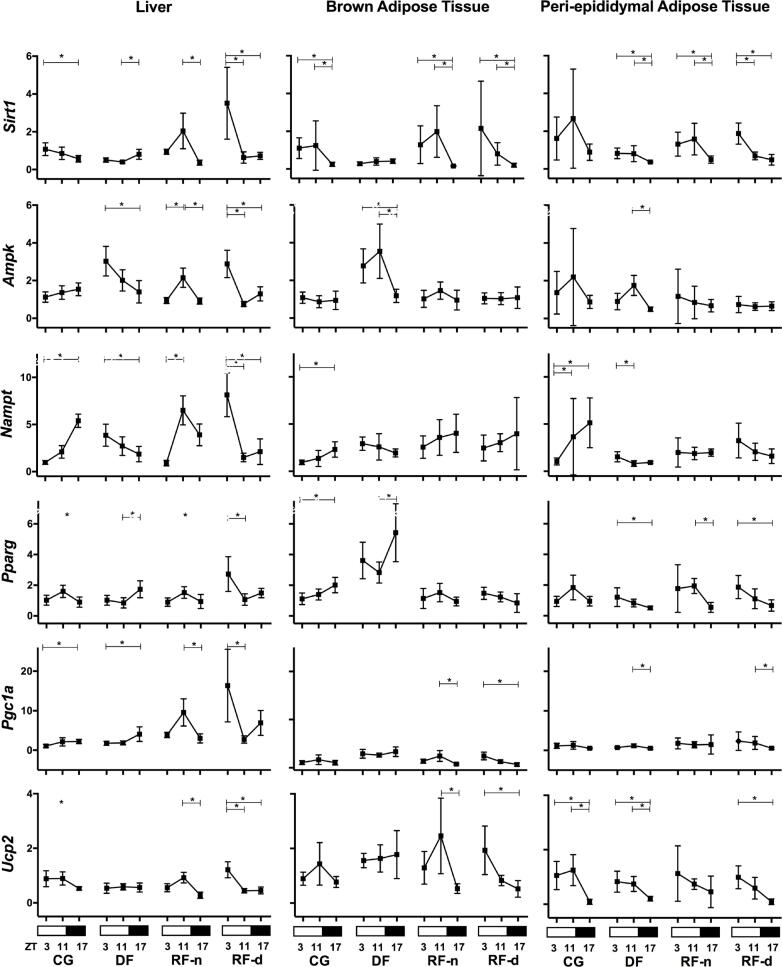
Relative expression (2^-ΔΔCt^) of energy-sensing genes ( *Sirt1* , *Ampk* , and *Nampt* ) and lipid metabolism-related genes ( *Pparg* , *Pgc1a* , and *Ucp2* ) in the liver, BAT (brown adipose tissue), and PAT (peri-epididymal adipose tissue) of Control group (CG), Day-fed (DF), Restricted night-fed (RF-n), and Restricted day-fed (RF-d) rats at ZT3 (0900 h), ZT11 (1700 h), and ZT17 (2300 h).

Figure 3 shows the expression of energy-sensing and lipid metabolism-related genes in the liver, BAT, and PAT of all studied groups. In the liver of the CG, the expression of *Sirt1* (p = 0.03) and *Ucp2* (p = 0.02) was higher in the morning (ZT3) and lower at night (ZT17), in contrast to the higher expression of *Nampt* (p < 0.0001) and *Pgc1a* (p = 0.02) that occurred at night. A higher expression of *Pparg* was observed in the afternoon (p = 0.03), whereas no differential expression of *Ampk* was observed among any time points. In the RF-n group, differently from the CG, higher expressions of *Sirt1* (p < 0.0001), *Ampk* (p = 0.001), *Pparg* (p = 0.04), *Pgc1a* (p = 0.0007), *Ucp2* (p < 0.0001), and *Nampt* (p < 0.0001) were observed in the afternoon (ZT11). Compared with RF-n, the RF-d group exhibited an inverse pattern, with lower expression at ZT11 for *Sirt1* (p < 0.0001), *Ampk* (p < 0.0001), *Pparg* (p < 0.0001), *Pgc1a* (p < 0.0001), *Ucp2* (p < 0.0001), and *Nampt* (p = 0.0007). The DF group showed higher expression of *Sirt1* (p = 0.0001), *Pparg* (p = 0.004), and *Pgc1a* (p = 0.01) at ZT17 and *Ampk* (p = 0.0005) and *Nampt* (p = 0.01) at ZT3, whereas *Ucp2* exhibited no differential expression among any time points. Thus, in the liver, the food availability during the 12 hours of the light phase (DF) inverted the profile of the energy-sensing and lipid metabolism-related genes observed in controls, except for *Pgc1a* . In addition, the calorie restriction (RF-d and RF-n) induced an increased expression of these genes, mainly in anticipation of food availability.

* p < 0.05

In the BAT, the CG exhibited higher expression of *Sirt1* at ZT11 (p = 0.002) and higher expression of *Nampt* (p = 0.02) and *Pparg* (p = 0.003) at ZT17, whereas *Ampk* , *Pgc1a,* and *Ucp2* exhibited no differential expression among any time points. The RF-n group also exhibited higher expression of *Sirt1* (p = 0.0001) at ZT11; the same pattern was observed for *Pgc1a* (p = 0.01) and *Ucp2* (p = 0.008), whereas no differential expression among morning, afternoon, and night was observed for *Ampk* , *Nampt* , and *Pparg.* In the RF-d group, higher expression of *Sirt1* (p = 0.0002), *Pgc1a* (p < 0.0001), and *Ucp2* (p = 0.0001) occurred at ZT3, and no differential expression among time points was observed for *Ampk* , *Nampt* , and *Pparg* . The DF group showed higher expression of *Ampk* (p = 0.0001) at ZT11 and of *Pparg* (p = 0.02) at ZT17.

In the PAT, the CG exhibited no difference in gene expression of *Sirt1* , *Ampk* , *Pparg* , and *Pgc1a* among the time points. Lower expressions of *Nampt* (p = 0.0001) and *Ucp2* (p = 0.002) occurred at ZT3 and ZT17, respectively. The RF-n group presented no difference in the expression of *Ampk* , *Pgc1a, Nampt,* and *Ucp2* between morning, afternoon, and night. A higher expression of *Sirt1* (p = 0.0002) and *Pparg* (p = 0.003) were observed at ZT11. The RF-d group showed lower expression of *Sirt1* (p = 0.0002), *Pparg* (p = 0.004), *Pgc1a* (p = 0.02), and *Ucp2* (p < 0.0001) at ZT17, whereas no differential expression of *Ampk* and *Nampt* was observed among all time points. The DF group exhibited higher expression of *Sirt1* (p = 0.003), *Pparg* (p = 0.02), *Nampt* (p = 0.03), and *Ucp2* (p = 0.0008) at ZT3. *Ampk* (p < 0.0001) and *Pgc1a* (p = 0.01) exhibited higher expression at ZT11. In summary, in the adipose tissues, daytime feeding modified the *Sirt1* expression pattern, and calorie restriction increased its gene expression, preceding mealtime. Differently from our findings observed regarding calorie restriction in the liver, in adipose tissues, there was a concurrent expression pattern of *Sirt1* , *Pparg* , *Pgc1a* , and *Ucp2* but not of *Sirt1* , *Ampk,* and *Nampt* .

## DISCUSSION

**Figure 4:**
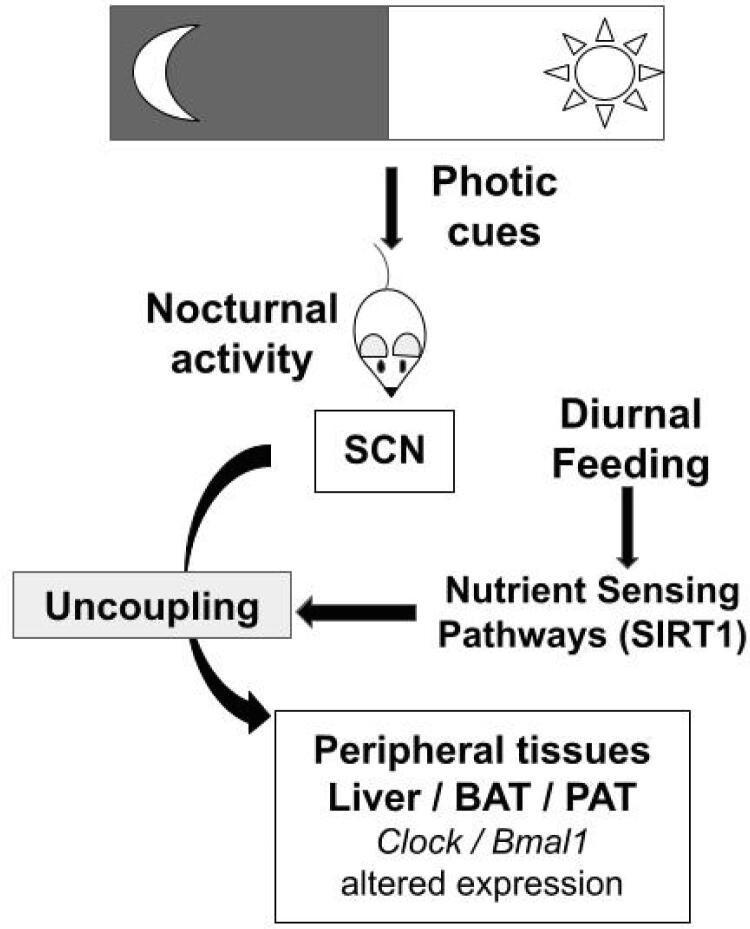
Schematic summary of study hypothesis and main results. Food restriction, in dissociation with rat nocturnal activity, alters the expression pattern of clock genes in peripheral tissues, such as liver and adipose tissues. These interactions may occur through *Sirt1* , a nutrient-sensing gene. BAT: brown adipose tissue; PAT: peri-epididymal adipose tissue; SCN: suprachiasmatic nucleus.

In the present study, we hypothesized that different feeding restriction patterns could reset clock genes ( *Clock* and *Bmal1* ) and lipid metabolism-involved genes ( *Pgc1a* , *Pparg* , *Ucp2* ) through nutrient-sensing-related genes ( *Sirt1* , *Ampk* , *Nampt* ). In our study, food restriction during the night preserved the expression pattern of clock genes in the liver, but there was a shift of this profile when the food access occurred in dissociation with the rat’s nocturnal activity, as illustrated in Figure 4 . In the adipose tissues, the shift phenomenon occurred at a lower magnitude. In addition, in the liver as well as in adipose tissues, food availability during the light phase inverted the profile of energy-sensing and lipid metabolism-related genes, whereas the calorie restriction induced an increased expression of these genes, mainly in anticipation of food availability.

Daytime feeding in murine is an important zeitgeber for peripheral clock genes and is capable of uncoupling the peripheral oscillators from the SCN ( 2 , 6 ). Previously, using the same protocol, we observed that restricted food availability led to decreased body weight, mostly due to the amount of food eaten and not the time of feeding. We also observed a pre-feeding corticosterone peak when animals were submitted to restricted food availability independently of the feeding time ( 16 ).

In the present study, our results demonstrate that in rats submitted to daytime feeding (RF-d or DF), independently of the amount of food eaten, the expression pattern of *Clock* and *Bmal1* was modified in the liver compared with control and RF-n groups, in which food intake was in accordance with rat nocturnal activities. These data are in accordance with a previous study evaluating daytime feeding in mice, in which an inverted phase of *Per1* , *Per2* , and *Dbp* clock genes was observed in the liver ( 18 ). In rats submitted to daytime feeding, higher expression of *Bmal1* at the end of the day (or the timing of feeding) has also been described, independently of feeding or fasting conditions ( 19 ). One recent study involving transcriptomic analysis indicated that, besides *Bmal1* and *Clock* , nearly all rhythmic transcripts exhibited a 12-hour phase shift in the liver of mice under diurnal feeding, called reverse-phase feeding, suggesting a dominant role of feeding on rhythmic phase regulation ( 20 ).

Similarly to the observed expression in the liver, higher *Bmal1* expression in the PAT was observed in the control and RF-n groups in the morning. *Bmal1* expression was modified exclusively by daytime feeding (DF and RF-d groups). However, in the BAT, both daytime feeding and calorie restriction at night (RF-n) modified *Bmal1* expression. Our data confirm previously reported higher *Bmal1* expression in the morning in brown and epididymal white adipose tissues from control animals and its phase shifting in daytime-fed animals ( 21 ). Furthermore, our data expand information about *Bmal1* phase shifting in adipose tissues from animals submitted to restricted feeding during a few hours of the light phase. Interestingly, *Bmal1* has been associated with regulation of brown and white adipose tissue differentiation ( 22 , 23 ).

In fact, the molecular mechanisms by which feeding affects the circadian clock remain not completely defined. Recently, Liu and cols. demonstrated that mechanistic target of rapamycin (mTOR) may modulate the hepatic circadian clock via tight junction protein 1 (TJP1). Upon feeding, mTOR phosphorylates TJP1, disrupting the TJP1/PER1 association and promoting PER1 nuclear translocation to inhibit the expression of CLOCK/BMAL1 target genes ( 24 ). In food-restricted rats, the food anticipatory activity was also associated with a significant reduction of liver glycogen levels, an increase of circulating free fatty acids and ketone bodies, and the occurrence of an oxidized cytoplasmic and mitochondrial redox state, which could cooperate to induce a new status of handling and distribution of nutrients by the liver upon food restriction ( 25 ).

Few studies have addressed the role of *Sirt1* and *Ampk* as possible candidates to entrain the peripheral clocks in response to feeding time ( 12 ). Our data support that *Sirt1* expression in the liver and in adipose tissues is modified by dietary restriction, with higher levels preceding mealtime. We also observed that *Ampk* and *Nampt* expressions in the liver were modified by both daytime feeding and dietary restriction. *Ampk* and *Nampt* expression patterns were concurrent with *Sirt1* only in the liver of animals submitted to intense calorie restriction. These findings reinforce the importance of SIRT1 as a nutrient-sensing molecule in regulating the circadian clock, specifically in the liver. It has been shown that mice treated with metformin, a commonly used drug whose mechanism of action has been linked to the activation of AMPK, exhibited a 3-h phase advance in liver clock genes ( *Per1* , *Clock* , *Bmal1,* and *Rora* and muscle *Bmal1* and *Rev-erba* ) ( 26 ). Furthermore, mice submitted to a high-fat diet exhibited decreased hepatic AMPK expression and activity, accompanied by disturbance of hepatic clock genes’ circadian rhythm ( 27 ). In accordance with our data, Chaix and cols. observed, in *Clock* mutant mice submitted to feeding time restriction, altered liver nicotinate and nicotinamide, two precursors of NAD+. Both are upstream of *Nampt,* the expression of which was increased in animals submitted to food restriction ( 5 ).

*Ampk* and *Nampt* presented no differential expression in restricted feeding groups in the BAT and PAT, suggesting that the metabolic changes evoked by food restriction in these tissues might not be mediated by the interaction between *Sirt1* and *Ampk* or *Nampt* , as suggested in the liver. In the BAT, other sirtuins, such as SIRT2 and SIRT3, may be relevant ( 28 ). Moreover, SIRT1 deacetylase activity can also vary in a circadian manner, regardless of its expression ( 13 ), which is a limitation in studies evaluating gene or protein expression. In addition, fasting conditions are associated with higher expression of *Sirt1* mRNA ( 19 , 29 ). Thus, the influence of fasting cannot be totally excluded in our study. Additionally, experimental animals might adapt to restricted levels of energy intake, mainly by a reduction in basal metabolic rate and in total heat production, with either reduction or no difference in spontaneous activity compared with ad libitum-fed rats. However, in the present study, spontaneous activity was not evaluated. Interestingly, a study on calorie restriction and weight loss in humans demonstrated an increase in SIRT1 expression in subcutaneous adipose tissue, concomitant with an increased glucose uptake in the BAT, which could indicate that the increase in SIRT1 could indirectly improve BAT metabolic efficiency upon weight loss ( 30 ).

We also observed a concurrent *Sirt1* expression pattern with the expression patterns of *Pparg* , *Pgc1a* , and *Ucp2* in liver and adipose tissues in different feeding schedules, reinforcing the control exerted by Sirt1 protein in lipid metabolism. Upon fasting, SIRT1 is recruited to the promoters of PPARG target genes in adipose tissue, counteracting this important adipogenesis regulator and promoting mobilization of fat stores during food deprivation ( 31 ). SIRT1 has also been identified as a PPARG deacetylase, whose effects could mimic outcomes of ligand-dependent PPARG activation ( 32 ). Indeed, resveratrol, through activation of the AMPK/SIRT1, prevents liver fat accumulation induced by high-fat and high-sucrose diets in rats by increasing fatty acid oxidation and decreasing lipogenesis ( 33 ). In addition, SIRT1 liver-specific deleted or knocked-down mice exhibited impaired fatty acid oxidation and increased hepatic free fatty acids ( 34 , 35 ). In myotubes, elevated SITR1 promotes deacetylation and activation of PGC1A, resulting in increased expression of molecules involved in mitochondrial biogenesis, such as UCP2 ( 36 ).

In conclusion, food restriction in dissociation with rat nocturnal activity alters the expression pattern of clock genes mainly in the liver. In addition, food availability during the 12 hours of the light phase inverted the profile of the energy-sensing and lipid metabolism-related genes, whereas the calorie restriction induced an increased expression of these genes in anticipation of food availability, also mainly in the liver. These interactions in liver and adipose tissues may occur through *Sirt1* , but the interaction with *Ampk* and *Nampt* might also be involved in the liver. Thus, dietary restriction resets clock and lipid metabolism-related genes in liver and adipose tissues through nutrient-sensing genes.

## SUPPLEMENTARY

**Supplementary Table 1:** Relative expression of clock genes, energy-sensing and metabolism-related genes in rat liver on restricted feeding schedules

Gene	Liver											
	CG			DF			RF-n			RF-d		
	ZT3	ZT11	ZT17	ZT3	ZT11	ZT17	ZT3	ZT11	ZT17	ZT3	ZT11	ZT17
*Clock*	1.1 ± 0.1	0.5 ± 0.1	1.1 ± 0.1	0.5 ± 0.1	0.6 ± 0.1	1.8 ± 0.3	0.9 ± 0.1	0.4 ± 0.1	1.0 ± 0.1	0.7 ± 0.2	0.7 ± 0.1	1.0 ± 0.1
	1.0	0.5	1.2	0.5	0.6	1.8	0.9	0.4	1.0	0.7	0.7	1.0
	[1.0-1.2]	[0.4-0.6]	[1.0-1.3]	[0.4-0.6]	[0.5-0.8]	[1.6-2.1]	[0.8-1.0]	[0.3-0.5]	[0.9-1.0]	[0.6-0.9]	[0.6-0.8]	[0.9-1.0]
*Bmal1*	1.0 ± 0.3	0.03 ± 0.01	0.4 ± 0.1	0.03 ± 0.01	0.5 ± 0.1	0.9 ± 0.3	0.8 ± 0.1	0.02 ± 0.01	0.6 ± 0.1	0.04 ± 0.02	0.8 ± 0.1	1.6 ± 0.2
	1.0	0.03	0.4	0.03	0.4	0.9	0.8	0.01	0.6	0.04	0.8	1.6
	[0.8-1.3]	[0.03-0.04]	[0.3-0.5]	[0.03-0.04]	[0.4-0.5]	[0.7-1.0]	[0.7-0.9]	[0.01-0.02]	[0.6-0.7]	[0.02-0.04]	[0.7-0.9]	[1.5-1.8]
*Silt1*	1.1 ± 0.3	0.9 ± 0.3	0.6 ± 0.2	0.5 ± 0.1	0.4 ± 0.1	0.8 ± 0.3	0.9 ± 0.1	2.0 ± 0.9	0.4 ± 0.1	3.5 ± 1.9	0.6 ± 0.3	0.7 ± 0.2
	1.0	0.8	0.6	0.5	0.4	0.8	1.0	1.8	0.3	3.0	0.6	0.7
	[0.8-1.4]	[0.6-1.2]	[0.4-0.7]	[0.4-0.6]	[0.4-0.5]	[0.6-0.9]	[0.8-1.0]	[1.4-2.8]	[0.3-0.5]	[2.0-4.7]	[0.4-0.9]	[0.6-0.8]
*Ampk*	1.1 ± 0.3	1.4 ± 0.4	1.5 ± 0.3	3.0 ± 0.8	2.0 ± 0.6	1.4 ± 0.6	0.9 ± 0.2	2.2 ± 0.5	0.9 ± 0.2	2.9 ± 0.7	0.8 ± 0.1	1.3 ± 0.4
	1.0	1.3	1.6	3.0	1.9	1.4	1.0	2.1	0.9	3.1	0.7	1.3
	[0.9-1.4]	[1.1-1.6]	[1.2-1.8]	[2.3-3.5]	[1.6-2.6]	[0.8-1.9]	[0.8-1.1]	[1.7-2.6]	[0.8-1.0]	[2.2-3.4]	[0.6-0.9]	[1.0-1.6]
*Nampt*	1.0 ± 0.2	2.1 ± 0.7	5.4 ± 0.7	3.9 ± 1.2	2.7 ± 1.0	1.9 ± 0.8	0.9 ± 0.3	6.5 ± 1.5	3.9 ± 1.1	8.2 ± 2.3	1.5 ± 0.5	2.1 ± 1.4
	1.0	2.1	5.6	3.8	2.4	1.6	0.8	7.0	3.7	8.3	1.4	1.7
	[0.8-1.2]	[1.6-2.5]	[4.7-6.0]	[3.5-4.5]	[1.7-3.6]	[1.1-2.8]	[0.7-1.1]	[5.1-7.7]	[3.0-4.8]	[6.0-10.2]	[1.2-1.7]	[1.1-3.3]
*Pparg*	1.0 ± 0.3	1.6 ± 0.4	0.9 ± 0.4	1.0 ± 0.3	0.9 ± 0.3	1.7 ± 0.5	0.9 ± 0.3	1.5 ± 0.4	0.9 ± 0.5	2.7 ± 1.1	1.1 ± 0.4	1.5 ± 0.3
	1.0	1.7	1.0	1.0	0.7	1.6	0.9	1.5	0.7	2.6	1.2	1.4
	[0.7-1.3]	[1.4-1.8]	[0.6-1.2]	[0.8-1.4]	[0.7-1.0]	[1.2-2.2]	[0.7-1.1]	[1.2-1.8]	[0.6-1.3]	[2.0-3.3]	[0.6-1.2]	[1.2-1.8]
*Pgc1a*	1.1 ± 0.4	2.1 ± 1.1	2.2 ± 0.6	1.8 ± 0.5	1.9 ± 0.5	4.0 ± 1.8	3.9 ± 0.7	9.6 ± 3.4	3.0 ± 1.2	16.4 ± 9.2	2.7 ± 0.9	7.0 ± 3.1
	1.0	1.6	2.0	1.7	1.7	4.3	4.1	10.1	2.7	15.5	2.5	7.3
	[0.7-1.5]	[1.4-3.0]	[1.8-2.7]	[1.4-2.4]	[1.6-2.5]	[2.1-5.6]	[3.1-4.5]	[6.6-12.2]	[2.0-4.1]	[7.5-24.1]	[2.2-2.9]	[5.1-8.0]
*Ucp2*	0.9 ± 0.3	0.9 ± 0.2	0.5 ± 0.06	0.5 ± 0.2	0.6 ± 0.1	0.6 ± 0.2	0.6 ± 0.2	0.9 ± 0.2	0.3 ± 0.1	1.2 ± 0.3	0.4 ± 0.1	0.4 ± 0.1
	1.0	0.9	0.5	0.5	0.6	0.5	0.6	1.0	0.3	1.1	0.4	0.4
	[0.6-1.1]	[0.7-1.1]	[0.5-0.6]	[0.4-0.6]	[0.5-0.7]	[0.4-0.7]	[0.4-0.7]	[0.8-1.1]	[0.2-0.4]	[0.9-1.6]	[0.4-0.5]	[0.3-0.6]

Data (2^-ΔΔCt^) are shown as mean ±; standard deviation, median and [interquartile interval]. CG: control group, DF: day-fed, RF-n: restricted night-fed, and RF-d: restricted day-fed. ZT3, ZT11 and ZT17: Zeitgeber times 3, 11 and 17, corresponding to 0900, 1700, and 2300h, respectively.

**Supplementary Table 2:** Relative expression of clock genes, energy-sensing and metabolism-related genes in rat brown adipose tissue on restricted feeding schedules

Gene	Brown adipose tissue											
	CG			DF			RF-n			RF-d		
	ZT3	ZT11	ZT17	ZT3	ZT11	ZT17	ZT3	ZT11	ZT17	ZT3	ZT11	ZT17
*Clock*	1.0 ± 0.2	1.0 ± 0.3	1.0 ± 0.1	1.0 ± 0.2	1.0 ± 0.7	1.2 ± 0.2	1.0 ± 0.7	0.6 ± 0.2	0.8 ± 0.1	1.2 ± 0.6	1.0 ± 0.5	0.8 ± 0.2
	1.0	0.9	1.0	1.0	0.9	1.3	0.6	0.5	0.8	0.9	0.8	0.7
	[0.8-1.2]	[0.8-1.2]	[0.9-1.1]	[0.7-1.1]	[0.7-1.0]	[1.0-1.4]	[0.5-1.6]	[0.5-0.8]	[0.7-1.0]	[0.7-1.8]	[0.7-1.6]	[0.6-0.9]
*Bmal1*	1.0 ± 0.3	0.1 ± 0.08	0.3 ± 0.1	1.0 ± 0.7	0.7 ± 0.3	0.4 ± 0.2	0.4 ± 0.2	0.2 ± 0.2	0.2 ± 0.2	0.9 ± 0.5	1.4 ± 0.5	0.1 ± 0.1
	1.0	0.1	0.3	1.0	0.6	0.3	0.5	0.2	0.2	0.8	1.2	0.07
	[0.8-1.3]	[0.06-0.2]	[0.1-0.4]	[0.4-1.6]	[0.4-0.8]	[0.2-0.5]	[0.1-0.6]	[0.07-0.4]	[0.08-0.4]	[0.6-1.0]	[0.9-1.9]	[0.03-0.1]
*Silt1*	1.1 ± 0.5	1.2 ± 1.3	0.2 ± 0.1	0.3 ± 0.1	0.4 ± 0.2	0.4 ± 0.1	1.3 ± 1.0	2.0 ± 1.4	0.2 ± 0.02	2.2 ± 2.5	0.8 ± 0.6	0.2 ± 0.1
	1.0	0.6	0.2	0.3	0.3	0.4	1.1	2.1	0.2	1.0	0.5	0.2
	[0.7-1.7]	[0.4-2.4]	[0.2-0.3]	[0.2-0.3]	[0.2-0.5]	[0.4-0.5]	[0.3-2.3]	[0.6-3.3]	[0.1-0.2]	[0.5-4.2]	[0.3-1.1]	[0.1-0.3]
*Ampk*	1.1 ± 0.3	0.9 ± 0.3	0.9 ± 0.5	2.8 ± 0.9	3.5 ± 1.4	1.2 ± 0.3	1.0 ± 0.5	1.5 ± 0.5	1.0 ± 0.5	1.0 ± 0.3	1.0 ± 0.3	1.1 ± 0.6
	1.0	0.9	0.9	2.9	3.7	1.1	1.0	1.6	1.0	1.1	1.0	1.1
	[0.9-1.2]	[0.6-1.2]	[0.5-1.4]	[1.9-3.4]	[1.8-5.1]	[1.0-1.4]	[0.6-1.4]	[1.0-1.8]	[0.3-1.4]	[0.8-1.3]	[0.8-1.3]	[0.5-1.5]
*Nampt*	1.0 ± 0.2	1.4 ± 0.8	2.3 ± 0.8	2.9 ± 0.7	2.6 ± 1.4	1.9 ± 0.4	2.6 ± 1.2	3.6 ± 1.9	4.0 ± 2.0	2.5 ± 1.4	3.0 ± 1.0	4.0 ± 3.8
	1.0	1.3	2.1	3.1	2.5	2.0	2.4	4.6	3.6	1.9	3.1	2.3
	[0.8-1.1]	[0.5-2.2]	[1.8-3.0]	[2.2-3.4]	[1.4-3.8]	[1.5-2.2]	[1.6-3.7]	[1.5-5.1]	[2.3-67]	[1.5-4.1]	[2.3-4.0]	[1.7-5.2]
*Pparg*	1.1 ± 0.4	1.4 ± 0.4	2.0 ± 0.5	3.6 ± 1.2	2.8 ± 0.7	5.4 ± 1.9	1.1 ± 0.7	1.5 ± 0.6	0.9 ± 0.3	1.5 ± 0.4	1.2 ± 0.3	0.8 ± 0.6
	1.0	1.6	1.9	3.2	2.6	5.0	1.0	1.6	0.8	1.4	1.3	0.7
	[0.8-1.4]	[1.0-1.7]	[1.7-2.3]	[3.0-4.8]	[2.1-3.6]	[5.4-6.9]	[0.7-1.7]	[1.0-2.0]	[0.7-1.3]	[1.1-1.8]	[0.9-1.4]	[0.3-1.2]
*Pgc1a*	1.1 ± 0.4	1.7 ± 1.0	1.1 ± 0.5	2.9 ± 0.9	2.6 ± 0.4	3.3 ± 1.0	1.4 ± 0.4	2.4 ± 1.1	0.8 ± 0.2	2.4 ± 0.8	1.3 ± 0.3	0.7 ± 0.3
	1.0	1.4	0.9	3.1	2.5	3.4	1.5	3.0	0.7	2.1	1.3	0.6
	[0.8-1.4]	[1.1-2.4]	[0.8-1.3]	[2.0-3.7]	[2.3-3.0]	[2.5-4.0]	[0.9-1.7]	[1.3-3.3]	[0.6-0.9]	[1.7-3.4]	[1.0-1.5]	[0.4-1.0]
*Ucp2*	0.9 ± 0.2	1.4 ± 0.8	0.8 ± 0.2	1.6 ± 0.3	1.6 ± 0.5	1.8 ± 0.9	1.3 ± 0.6	2.5 ± 1.4	0.6 ± 0.2	1.9 ± 0.9	0.8 ± 0.2	0.5 ± 0.3
	1.0	1.5	0.8	1.5	1.6	1.6	1.1	2.4	0.6	1.7	0.8	0.4
	[0.6-1.1]	[0.7-2.1]	[0.6-1.0]	[1.4-1.8]	[1.1-2.2]	[1.0-2.2]	[0.8-1.9]	[1.2-3.7]	[0.3-0.7]	[1.5-2.3]	[0.7-1.0]	[0.3-0.7]

Data (2^-ΔΔCt^) are shown as mean ±; standard deviation, median and [interquartile interval]. CG: control group, DF: day-fed, RF-n: restricted night-fed, and RF-d: restricted day-fed. ZT3, ZT11 and ZT17: Zeitgeber times 3, 11 and 17, corresponding to 0900, 1700, and 2300h, respectively.

**Supplementary Table 3:** Relative expression of energy-sensing and metabolism-related genes in rat peri-epididymal adipose tissue on restricted feeding schedules

Gene	Peri-epididymal adipose tissue											
	CG			DF			RF-n			RF-d		
	ZT3	ZT11	ZT17	ZT3	ZT11	ZT17	ZT3	ZT11	ZT17	ZT3	ZT11	ZT17
*Clock*	1.0 ± 0.5	0.8 ± 0.3	1.0 ± 0.2	0.5 ± 0.1	0.5 ± 0.2	0.4 ± 0.1	1.0 ± 0.3	0.3 ± 0.1	0.5 ± 0.1	0.8 ± 0.2	0.8 ± 0.6	0.4 ± 0.1
	1.0	0.8	0.9	0.5	0.5	0.4	0.9	0.3	0.5	0.8	0.6	0.4
	[0.5-1.4]	[0.6-0.9]	[0.8 ± 1.2]	[0.4-0.7]	[0.4-0.7]	[0.4-0.5]	[0.8-1.3]	[0.2-0.4]	[0.4-0.6]	[0.6-1.0]	[0.4-1.6]	[0.3-0.6]
*Bmal1*	1.0 ± 0.3	0.06 ± 0.04	0.2 ± 0.1	0.2 ± 0.07	0.2 ± 0.08	0.1 ± 0.06	0.3 ± 0.1	0.04 ± 0.02	0.4 ± 0.1	0.2 ± 0.07	1.0 ± 0.3	0.2 ± 0.1
	1.0	0.07	0.1	0.2	0.2	0.1	0.3	0.04	0.4	0.2	1.0	0.1
	[0.7-1.2]	[0.03-0.1]	[0.1-0.3]	[0.1-0.2]	[0.1-0.3]	[0.07-0.2]	[0.2-0.4]	[0.02-0.05]	[0.3-0.5]	[0.1-0.2]	[0.8-1.1]	[0.07-0.3]
*Silt1*	1.6 ± 1.1	2.7 ± 2.6	0.9 ± 0.4	0.8 ± 0.3	0.8 ± 0.4	0.4 ± 0.04	1.3 ± 0.6	1.6 ± 0.8	0.5 ± 0.2	1.9 ± 0.6	0.7 ± 0.2	0.5 ± 0.3
	1.0	1.7	0.9	0.8	0.7	0.4	1.1	1.1	0.4	2.1	0.8	0.4
	[0.9-2.8]	[0.8-4.6]	[0.5-1.3]	[0.5-1.1]	[0.5-1.0]	[0.3-0.4]	[0.8-1.9]	[1.0-2.4]	[0.4-0.7]	[1.4-2.2]	[0.5-0.8]	[0.3-0.8]
*Ampk*	1.4 ± 1.1	2.2 ± 2.6	0.9 ± 0.3	0.9 ± 0.4	1.8 ± 0.5	0.5 ± 0.1	1.2 ± 1.4	0.8 ± 0.9	0.7 ± 0.3	0.7 ± 0.4	0.6 ± 0.2	0.6 ± 0.2
	1.0	1.3	0.9	0.9	1.6	0.5	0.6	0.5	0.7	0.6	0.6	0.6
	[0.7-2.0]	[0.7-3.4]	[0.5-1.2]	[0.5-1.3]	[1.4-2.3]	[0.4-0.6]	[0.5-1.6]	[0.4-1.5]	[0.6-0.7]	[0.4-1.1]	[0.5-0.8]	[0.5-0.8]
*Nampt*	1.0 ± 0.4	3.7 ± 4.0	5.1 ± 2.6	1.5 ± 0.5	0.8 ± 0.3	0.9 ± 0.1	2.0 ± 1.5	1.9 ± 0.7	2.0 ± 0.4	3.3 ± 1.8	2.1 ± 0.9	1.6 ± 0.8
	1.0	2.3	4.2	1.3	1.0	0.9	1.5	1.6	2.1	2.4	2.2	1.4
	[0.8-1.4]	[1.7-4.7]	[3.2-8.1]	[1.1 -2.1]	[0.5-1.0]	[0.8-1.1]	[1.1-2.6]	[1.4-2.5]	[1.7-2.2]	[1.4-5.1]	[1.5-2.4]	[1.2-1.9]
*Pparg*	0.9 ± 0.3	1.8 ± 0.8	0.9 ± 0.3	1.2 ± 0.6	0.8 ± 0.3	0.5 ± 0.1	1.8 ± 1.6	1.9 ± 0.5	0.5 ± 0.3	1.9 ± 0.8	1.1 ± 0.6	0.7 ± 0.4
	1.0	1.9	0.9	1.1	0.8	0.5	1.7	2.2	0.6	1.6	1.0	0.5
	[0.5-1.3]	[1.0-2.6]	[0.8-1.1]	[0.7-1.8]	[0.6-1.0]	[0.4-0.6]	[0.6-2.5]	[1.5-2.3]	[0.2-0.9]	[1.4-2.3]	[0.6-1.7]	[0.4-1.0]
*Pgc1a*	1.1 ± 0.7	1.3 ± 1.0	0.5 ± 0.2	0.7 ± 0.2	1.2 ± 0.4	0.5 ± 0.2	1.8 ± 1.4	1.4 ± 0.8	1.5 ± 2.4	2.3 ± 2.3	1.9 ± 1.6	0.5 ± 0.2
	1.0	1.2	0.6	0.7	1.3	0.5	1.2	1.5	0.7	2.1	1.2	0.4
	[0.8-1.6]	[0.4-2.0]	[0.4-0.6]	[0.6-0.9]	[0.9-1.5]	[0.3-0.6]	[0.8-3.0]	[0.7-2.1]	[0.4-1.0]	[0.7-2.5]	[0.9-2.2]	[0.3-0.8]
*Ucp2*	1.1 ± 0.5	1.3 ± 0.6	0.1 ± 0.1	0.8 ± 0.4	0.7 ± 0.3	0.2 ± 0.1	1.1 ± 1.0	0.7 ± 0.2	0.4 ± 0.6	1.0 ± 0.4	0.6 ± 0.4	0.1 ± 0.1
	1.0	1.1	0.04	0.7	0.8	0.2	0.6	0.8	0.2	1.0	0.4	0.05
	[0.6-1.6]	[0.8-1.7]	[0.02-0.2]	[0.5-1.2]	[0.5-1.0]	[0.1-0.3]	[0.4-2.3]	[0.5-0.9]	[0.1-0.9]	[0.6-1.4]	[0.3-0.7]	[0.02-0.2]

Data (2^-ΔΔCt^) are shown as mean ± standard deviation, median and [interquartile interval]. CG: control group, DF: day-fed, RF-n: restricted night-fed, and RF-d: restricted day-fed. ZT3, ZT11 and ZT17: Zeitgeber times 3, 11 and 17, corresponding to 0900, 1700, and 2300h, respectively.
